# Formative research to adapt the ‘Diabetes Prevention Program- Power to Prevent’ for implementation in Bamako, Mali

**DOI:** 10.1186/s12913-023-10515-6

**Published:** 2024-01-11

**Authors:** Lancina Doumbia, Sally Findley, Hamidou Oumar Ba, Bonkana Maiga, Aissata Ba, Rokiatou Koné Béréthé, Hadja Madjè Sangaré, S Patrick Kachur, Stéphane Besançon, Seydou Doumbia

**Affiliations:** 1 University Clinical Research Center, Université des Sciences, des Techniques et des Technologies de Bamako, Point-G, Bamako, Bamako, Po Box 5445, Mali; 2 Mailman School of Public Health, Columbia University, New York, NY USA; 3 ONG Santé Diabète, Bamako, Mali

**Keywords:** Diabetes in Africa, Diabetes prevention and management, Community health workers, DPP adaptation, Mali

## Abstract

**Background:**

There are few community-level behaviors change interventions for reducing diabetes and hypertension risk in Africa, despite increasing cases of type 2 diabetes and cardiovascular diseases. Thus, this study was designed to adapt the United States Centers for Disease Control and Prevention’s “Diabetes Prevention Program Power to Prevent” (DPP-P2P) for use in low-income urban communities of Bamako, Mali.

**Methods:**

Feedback was elicited on an initial French PowerPoint adaptation of the DPP-P2P session guidelines from stakeholders at the ministry of health, organizational partners, and medical care providers. Two community health centers in districts with high levels of diabetes or hypertension were selected to assist in developing the Malian adaptation. Focus groups were conducted with 19 community health workers (CHWs) of these centers. Based on feedback from these discussions, more graphics, demonstrations, and role plays were added to the PowerPoint presentations. The 19 CHWs piloted the proposed 12 sessions with 45 persons with diabetes or at-risk patients over a one-month period. Feedback discussions were conducted after each session, and changes in dietary and exercise habits were assessed pre and post participation in the program. This feedback contributed to finalization of a 14-session sequence.

**Results:**

The DPP-P2P session guidelines were adapted for use by low-literacy CHWs, converting the written English guidelines into French PowerPoint presentations with extensive use of pictures, role plays and group discussions to introduce diabetes, diet, and exercise concepts appropriately for the Bamako context. CHWs recommendations for a strong family-oriented program led to expanded sessions on eliciting support from all adults in the household. The 45 participants in the pilot adaptation were enthusiastic about the program. At the end of the program, there were significant increases in the frequency of daily exercise, efforts to limit fat intake, and goals for more healthy diets and exercise levels.

**Conclusion:**

This study documents how an iterative process of developing the DPP-P2P adaptation led to the development of a culturally appropriate set of materials welcomed by participants and having promise for reaching the low-income, low-literacy population with or at risk for diabetes in Bamako, Mali.

**Supplementary Information:**

The online version contains supplementary material available at 10.1186/s12913-023-10515-6.

## Background

In recent decades, in sub-Saharan Africa, the prevalence of diabetes and cardiovascular disease (CVD) increased by about 20% [1–3], especially among women living in urban areas [4]. In urban Mali, hypertension prevalence varies between 21% and 39%, [5, 6] and diabetes prevalence is estimated at 11% [7], both likely to be underestimates due to many undiagnosed or diagnosed without access to care [8–11]. These increases are associated with the nutritional transition in the rapidly growing cities, where there is increased consumption of obesogenic foods along with a more sedentary lifestyle [12]. In urban Mali, processed foods now constitute 60% of the diet and oils 7%, both higher shares than in rural Mali [13].

In Mali, diabetes care in Bamako is provided by the national center for diabetes care, and it is primarily medical, providing limited counseling on necessary lifestyle changes to support management [14, 15]. While there is evidence for supporting both prevention and control of diabetes and hypertension by changes to diet, physical activity, smoking, and alcohol consumption [16–19], none of the programs promoting these changes are available in Mali, despite evidence of their effectiveness in other countries [19–24].

In the United States (US), the National Diabetes Education Program (NDEP) of the Centers for Disease Control and Prevention (CDC) developed programs to support lifestyle behavior changes for improved self-management and prevention of diabetes, incorporating the communication/persuasion and transtheoretical models for behavior change [25, 26]. The NDEP “Diabetes Prevention Program Power to Prevent program” (DPP-P2P) consists of 12 educational sessions led by community health workers (CHWs) who facilitate the adoption of dietary behavior changes and physical activity using a Small Steps, Big Rewards approach [27], now proven effective in reducing diabetes risk in more than 40 different communities [19, 28, 29].

To date, there are limited adaptations of the DPP-P2P program for low-income countries [30, 31], with only one in progress in Africa, in South Africa [32]. The DPP-P2P requires substantial modification to account for the large cultural and contextual differences between urban West Africa and the US. In addition, the CHWs in West Africa typically have low levels of education, making it difficult for them to deliver a program based on printed instructional materials. Therefore, the objectives of this pilot study were: (1) to use a participatory approach to adapt the DPP-P2P sessions for delivery by CHWs in the Malian context, and (2) to obtain feedback on the adapted session and assess their potential for promoting behavioral change with potential participants.

## Methods

### Study site

Bamako, the capital city of Mali, had a population of about 2.5 million inhabitants in 2019 [33] with one of the highest growth rates in Africa, [34] visible in the city’s extension of informal settlements in peri-urban areas [35]. We invited two peri-urban community health centers (CSCOM for “Centre de Santé Communautaire”) to assist in developing the adaptation, selected because their districts have relatively high rates of diabetes and hypertension. Koulouba and Taliko CSCOMs serve 31,278 and 41,684 inhabitants, respectively. At each CSCOM, the medical officer was invited to support the study by assisting in the provision of space for the planned group sessions and in preparation of a list of patients with or at risk for diabetes seen at the CSCOM.

### Formative adaptation process

We used an iterative, participatory approach to adapt the 12 DPP-P2P sessions for use in Bamako [36] with: (1) Initial translation of the 12 DPP-P2P sessions into 12 DPP-Bamako sessions by the multi-disciplinary research team; (2) Feedback from stakeholders at the ministry, organizational partners, and medical care providers; (3) Focus groups with CHWs; (4) Further tailoring to the group presentations; (5) Pilot study with 45 participants diagnosed with or at risk for diabetes to obtain feedback on the adaptation. (6) Finalization of adaptation of the DPP-P2P for use in Bamako.

### Feedback focus group methods

Two one-hour focus group discussions with a total of 19 CHWs from the two selected CSCOMs were conducted in the lingua franca of Bamako, Bambara. These discussions covered five themes: (1) experience and knowledge of diabetes; (2) interest in a program supporting lifestyle change; (3) understanding about working in group; (4) perception of issues associated with making lifestyle changes; (5) suggestions for what others might need to help them change their lifestyle. Audio recordings of the discussions were professionally transcribed from Bambara into French and then analyzed using thematic content analysis, identifying key phrases or words using Microsoft Word. A similar methodology was used for the focus groups conducted with the two groups of 45 participants at the two CSCOMs at the end of their participation in the pilot of the “Programme de Prevention du Diabète au Mali” (PPD-Mali) adaptation. An English language version of the focus group discussions is available as a supplementary file *1*.

### Pilot study with potential participants

#### Pilot study sample

A quasi-experimental pre and post-test was designed to obtain participant feedback and assess the potential of the adapted sessions for promoting diet and exercise behavior changes. Participants invited by CHWs to a meeting to learn about the pilot study, were chosen among the patients known to be diagnosed with diabetes, obesity, or hypertension, on the list provided by the medical officers of the two CSCOM. Forty-five individuals (mainly female, average age 49) consented to participate in the pilot study. Over half (26 participants) had been diagnosed with diabetes, 32 had hypertension, and 13 had neither chronic disease diagnosis. They had been diagnosed an average of 10 year ago, and of those with diabetes, only half reported being on insulin.

#### Pilot study implementation

The CHWs were trained to assist the researchers in facilitating the sessions with the participants. Sessions were conducted 3 times a week for 4 weeks and consisted of PowerPoint presentations, group discussions, and role plays. Sessions were conducted in Bambara. Participants were introduced to the first 3 sessions as one group, and then they were split based on their neighborhood proximity into two groups: a physical activity group and a diet group. Sessions were held on alternate weekdays, with the physical activity group receiving sessions 4, 5, and 6, and the diet group sessions 7, 8, and 9. All groups participated in sessions 10, 11, and 12. Each session lasted 3 h and began with a round table discussion with participants, to obtain feedback on both easiness and difficulties they encountered when applying lessons learned during the previous session. The session closed with participants providing verbal feedback on the session itself.

#### Pilot study feedback and behavioral change assessment

Participants’ pre-post changes in diet and physical activity behaviors were assessed by a 26-question, multiple choice survey administered before session 1 and after session 12. The pre-test also included questions about challenges they faced in changing their lifestyle behavior after being diagnosed with their condition. Research team members assisted illiterate participants in completing the surveys. Descriptive analysis of demographic information and pre- and post-test differences in activity and diet were assessed using Stata v. 15.1. Focus groups were conducted after the post-test survey to obtain overall feedback on the program’s acceptability and to elicit suggestions for further improvements to the adaptation.

### Research approvals

Support for the study was provided by two grants, from the non-profit HPI Institute and the National Institutes of Health (NIH) (R21 TW011736). All methods were performed in accordance with relevant guidelines and regulations. The pilot pre-post study protocol was approved by the Ethics Committee of the Faculty of Medicine and Dentistry and the Faculty of Pharmacy of the Université des Sciences, des Techniques et de Technologies de Bamako (USTTB) as an exempt study. Columbia University Institutional Review Board provided a non-research determination on behalf of US-based collaborators. Participants in the focus groups and feedback sessions provided oral informed consents.

## Results

### Initial adaptation by the multi-disciplinary research team

We reviewed the 12 DPP-P2P “Road to Health Toolkit” group sessions and supporting materials from the “Small Steps, Big Rewards to Prevent Type 2 Diabetes” to determine how they should be revised to be consistent with the cultural and economic context experienced by potential participants in Bamako. Most importantly, we needed to make the materials culturally relevant, featuring African participants eating African food and exercising in a typical urban African environment. For instance, most people in low-income peri-urban setting of Bamako have a low-literacy level, and even if literate there is little consumption of packaged foods which could contain nutritional content labels [13]. So, the sessions on label reading and calorie counting were not relevant. Instead of calorie counting, we adopted the concept of reporting portions [37, 38], emphasizing measurement and counting daily portions of rice and other starchy staples. A major change in approach also was needed to accommodate the social realities of shared food preparation and eating from a common bowl in Bamako households. Consistent with other studies showing the importance of families in promoting lifestyle changes for diabetes management [39, 40], we needed to build in strategies to gain the support of the male head of household and other adult women who may share cooking responsibilities. Organized exercise opportunities were also quite limited in Bamako, where residential areas of Bamako lack sidewalks or parks. Exercise recommendations had to be adapted as at-home workout routines or for the organization of small neighborhood dance or exercise groups. Because the CHWs leading the group sessions were themselves likely to have low levels of literacy, we needed to convert the DPP-P2P session guidelines into PowerPoint presentations in French, the official written language used in Mali, along with guidelines for subsequent verbal translation into Bambara (a local Manding language that serves as the lingua franca of Bamako). All presentations needed to include culturally appropriate pictures and graphics as much as possible. Finally, along with the original focus of the DPP-P2P on prevention of diabetes among those at risk, our adaptation also aimed to promote better self-management of diabetes through behavior change. While the initial diabetes education materials encouraged regular blood sugar testing and adherence to diabetes medications, no adaptations were made specifically for those who might be taking insulin.

### Stakeholders’ feedback

Over the course of next three months, we convened conversations and meetings with stakeholders at the neighborhood, regional, and national levels, to assess their interest in and support for the proposed adaptation of the DPP-P2P. The research team met with the medical officers and community health workers (CHWs) at the two participating CSCOMs. The medical officers could use their records to identify persons diagnosed with diabetes or hypertension, noting that they generally do not care for those with diabetes, who are referred to the national diabetes center for treatment or to the district hospital for urgent care. Each CSCOM had a roster of 8–10 CHWs whose involvement tended to be episodic for specific public health campaigns, and had not received any training for supporting those with chronic diseases. The medical officers were enthusiastic about having more engagement in the care of persons with diabetes or hypertension, as they felt that they currently had little connection to them except when patients experienced acute symptoms. They believed the CHWs would welcome such a program, which the CHWs themselves confirmed. We met with leaders of the national diabetes control and advocacy groups, who were very supportive of the proposed adaptation. They recommended incorporating diabetes peer educators into the team delivering the sessions, based on their positive results with this method [41, 42]. Finally, the national ministry division heads for non-communicable diseases and for community health acknowledged that they lacked any comparable ways to reach those at risk for or already diagnosed with diabetes and expressed enthusiasm for the proposed CHW-led diabetes educational program, which they could envision using nationally.

### CHW focus groups

#### Experience and knowledge of diabetes and hypertension

The CHWs had heard about hypertension and diabetes, and most knew someone with one of these diseases. They thought that recently more people were getting the diabetes or hypertension, even young people.

They say that hypertension is a disease for the elderly, but we notice today that there are young people who have hypertension which explains that it is not a disease of the elderly alone.

CHWs wanted a better understanding of the diseases and their risks, to educate and motivate people.

“It is said that if you practice sports or if you deprive yourself of salt in the evenings, you will not have hypertension. We do not understand this because very recently a ten-year-old child was diagnosed at our CSCOM with hypertension. We would like to know how these diseases arise, and what is the difference between those figures that go up and that go down when the doctor checks our pressure.”

#### Suggestions for behavior change

Some mentioned cutting down on salt, and then others added reducing sweets and sweet drinks.

We must try to diversify our meals by reducing the consumption of salt. Cooks tend to put lots of salt-concentrated bouillon cubes in the sauce.

Eliminating salty concentrated bouillon cubes was discussed in both groups, but reducing oil was mentioned only once. Cutting down rice consumption (portion size) was only mentioned once, and no one suggested increased consumption of fruits and vegetables.

Many believed that sports were supposed to help control both diabetes and hypertension. When asked about exercising, most thought this was only for young people. While a couple participants mentioned bicycling, most thought they would only walk, in their home courtyard or neighborhood.

#### How to motivate change

Involving family and close friends was mentioned several times in both groups. Participants said that making changes in diet for one individual was not easy.

It would be very good to involve a close person, it can be the spouse, or a child. The latter can remind you repeatedly while encouraging you.

They emphasized that any changes in diet must be for the whole family, necessarily involving the head of the household in the decision, as well as all women preparing food for the household. They said that reducing use of salt-concentrated bouillon cubes would be challenging, since they used the cubes to prepare tasty sauces on a limited budget. They underscored that the changes in diet could not be more expensive.

#### How to motivate participation in the program

They suggested that referral by a doctor might be necessary to motivate the partner or a family member to support them. However, they agreed that program participants still will need the CHWs to explain what needs to be done and visit them in their homes to provide support.

Findings from the CHW focus groups helped to identify where role plays would be more relevant than didactic presentations to explain behavior changes, as well as aspects of lifestyle change to be covered in the sessions.

### Further tailored adaptation of the DPP-P2P sessions and materials

Table 1 displays a grid showing the original group sessions for DPP-P2P and how they were adapted in this formative research.

**Table 1:** Initial Adaptation of the DPP-P2P sessions and related materials

Original DPP-P2P sessions	PPD-Mali (Mali DPP adaptation)	Major elements added or changed
1.Welcome to the DPP program	1.Welcome to PPD-Mali	Insert Santé Diabète Mali (SDM) “What is Diabetes? Simplify description of DPP outcomes Presentations and tools in French with Bamako pictures
2. Small Steps lead to Big Rewards (SSBR)	2. Small Steps for you and your spouse	Adapt SSBR game plan and food and activity trackers with local pictures SSBR flipchart changed to Halimatu & Amadou, SDM adaptation emphasizing how diet and activity affect health Add role plays illustrating spouses supporting each other
3.Getting started with your plan	3. Engage the family for social support	Adapt Benefits of Social Support module (# 10) for engaging family head Develop role plays to build confidence for introducing program to family head and co-wives.
4. Move More	4. Move More- At home and outside	Exercise demos from Malian exercise coach Videos of Senegalese women exercising and dancing
5. Reduce your calorie intake	5. Small steps toward healthy eating	Introduce the Benin healthy food hut [43] Include a recommended weekly menu, using the 5 common Malian sauces prepared with lower fat and calories to reduce their glycemic index [44]
6. Control temptation	6. Eat less but better	Adaptation of DPP session 9, with emphasis on portion sizes instead of calorie counting Role play estimating portions eaten from communal pot
7. Resolve problems	7. Resolving problems	Additional role plays and discussions on handling flagging motivation to change
8. Four keys to eating out	8. Five keys to healthy eating out	Remove items pertaining to reading menus Substitute recommendations on street food and buying food outside home and during family celebrations
	9. Drink water, not sodas	Add a separate module on drinking water, part of DPP session 9. Messages on reducing alcohol and beer, and restricting sweetened tea and juices
9. More volume, fewer calories	10. Healthy heart diet	Adaptation of DPP session 10 with a pictorial introduction to hypertension Add Benin healthy food hut [43] with stop light messaging about foods to avoid or “Stop” or to limit, “Caution” foods Emphasize reducing salt, bouillon cubes, and sugars
10. Have a healthy heart	11. How to keep your family motivated	Adaptation of DPP session 12, with more emphasis on motivation of spouse. Additional discussion on gaining support from family How to re-start the small steps, whether exercise or diet
11. Benefit from social support 12. Prepare for the long term	12. Looking backward to look forward	Adaptation of session 12 Celebration to thank each other, spouses, family, and CHWs

We retained the same basic sequence of sessions, but consistently made changes to enable presentation and use by peer educators and CHWs in Bamako. All sessions were guided by French PowerPoint presentations, designed to be delivered with translation into Bambara by the peer educator with support in group moderation and support from the CHW. The PowerPoint presentations emphasized key aspects of behavior change and conveyed concepts about diabetes, heart disease or calorie balance as much as possible with pictures. Sessions used the “Road to Health” interactive, participatory approach, but introduced these via the PowerPoint with group activities, with additional demonstrations and role plays providing opportunities for participants to practice talking about and making behavioral changes. All materials were transformed for use in the Bamako setting. For example, the DPP’s “Choose More Than 50 Ways to Prevent Type 2 Diabetes” was adapted to include realistic choices for people living in low-income communities in Bamako. Recognizing the low-literacy level and lack of any calorie labels, all diet recommendations and tracking tools were converted to portions, and portion size was taught in one of the sessions. Pictures were liberally added to the presentations, using local or culturally appropriate images showing Africans and African foods. Throughout the emphasis was placed on role plays to help participants have conversations with their family members to gain support for the changes they wanted to make.

### Participant feedback on the tailored DPP-P2P Sessions

#### Focus group feedback from participants

After one month of implementation of the adapted DPP-P2P sessions, the session attendance rates were over 96%, indicating a high level of interest in the program. At the concluding focus group, participants made helpful suggestions about the format and messages of the proposed adaptation of the DPP-P2P program for use in Bamako. Table 2 summarizes feedback from participants on the sessions. They stressed the importance of involving the family, especially all women preparing food for the household.

**Table 2:** Participant feedback on the adapted DPP-P2P sessions

Major themes	Participants’ feedback
*Importance of involving the family*	Participants learned how to reach out to family heads and other family members about modifying diet for the whole family and how to address criticisms. Managing these communications was considered a key for success. They agreed that it is important to continually remind oneself and others in the family that the objective is one’s own health. “It is necessary to involve all the cooks including co-wives in the diet change. They had to learn to continue, ignoring criticism so that they would not become discouraged.”
*Understanding how they can stay healthy* *More guidance on how to cook healthy within their budgets.*	Participants felt they better understood diabetes and what they can do to prevent or better care for themselves. Prevention for other members of the family was also something they had not understood. They liked and wanted more pictures, recipes, and demonstrations to guide them to cook more healthy sauces. They wanted advice on how to prepare healthy food without additional costs. They agreed that one could cut costs, such as salt-concentrated bouillon cubes, freeing this money to be used for vegetables to put in the sauces. In the session on eating out, they learned that instead of everyone purchasing lunch or snacks separately, they could pool the money and use it to buy fruit and vegetables for the whole family.
*How to control diet when eating from a shared bowl*	All agreed that changing the content of collective meals requires group solidarity, so that habits are changed for all and sustainably. They learned how to apply recommendations for portion size when meals are served from a shared bowl.
*Controlling diet is a continuous struggle but not impossible*	After participating in the sessions, they understood the importance of dietary diversity and balance. They were also pleased to learn about good alternatives to rice, such as the traditional millet pudding or le “tô malien,” which had been abandoned by many now living in Bamako.
*Changing physical activity can be done*	All agreed that this was much easier to do and had started immediately after the first three sessions. Some had also started walking, by themselves or with their husbands. At Taliko, they had started weekly walking/dancing groups. They suggested that the program should find a coach to mentor these groups in their exercise plans so that their activities are more effective. Several said they had already started to feel the good results of regular exercise.
*The Game Plan to track their changes in nutrition and exercise*	They liked the idea of a game plan, because it will help them continue striving for good results.
*Importance of the CHWs in supporting the behavior change*	Participants congratulated the CHWs who encouraged them in making changes. They were impressed that the CHWs had already started talking with their neighbors and friends about making changes.

#### Pre-post changes in behavior among participants

After one month of group session implementation, there were significant increases in those wanting to eat more healthy and be more active, and they had already begun to limit fat intake and increase their exercise levels. (Table 3).

**Table 3:** Pre-test vs. Post-test Healthy Behaviors and Goals

	Pre-test N = 34	Post-test N = 33	*p*
**Healthy Behavior**			
Limit fat intake	70.6%	97.0%	0.004
Limit how much I eat	61.8%	54.6%	0.549
Not active in past week	35.3%	12.1%	0.026
Active 3 + days in past week	32.3%	75.8%	< 0.001
**Individual goals**			
Lose weight	76.5%	90.0%	0.116
Eat more healthy	44.1%	71.4%	0.022
Be more active	41.2%	68.6%	0.022

These positive results after initial exposure to the DPP adaptation demonstrated that the program was acceptable to those who would be eligible to participate, and that there was a potential for participation to lead to positive changes in targeted lifestyle behaviors.

### Final adaptation of the DPP-P2P for use in Bamako, Mali

Based on the feedback from the pilot study participants, we proceeded with the following changes to the Malian adaptation of the DPP-P2P. First, we further reduced the written didactic components of the program, adding more pictures and discussion groups to convey concepts about diabetes risk and control. Second, we subdivided the sessions on moving more and diet change into two parts, to allow more time to introduce these concepts through demonstrations and activities. Third, we developed a pictorial cookbook for healthy preparations of the five most commonly prepared sauces in Mali, incorporating the findings from the nutritional analysis of the Food Composition Table for Mali [44] to develop instructions for their healthy preparation. These recipes used less fats, oil, salt, and bouillon cubes than in standard preparations, and both the fish and meat versions of these sauces were tested by five CHWs at Taliko who provided taste-tests to six randomly selected CSCOM visitors. Each stage of preparation was photographed for use in the cookbook. Local market costs for ingredients were provided in the cookbook, addressing the concerns of participants that the diet changes would be cost-neutral. Fourth, a professional exercise coach was hired to develop exercises to be used with participants. Working with the 10 Taliko CHWs, he showed them how to do 16 basic exercises at home. Each training session was video-taped and later edited to produce short videos with Bambara explanations, for use in the sessions promoting more exercise. Photos of these training sessions were incorporated into an exercise booklet with recommendations for an exercise menu with 3 levels of intensity. Fifth, so that participants could keep track of their portions of each type of food, we adapted the Game Plan daily food log to make it suitable for low-literacy participants, with pictures of the most common foods consumed in Bamako. Table 4 shows the final recommended organization for the Malian adaptation of the DPP-P2P. This final adaptation and accompanying materials were presented to the initial group of stakeholders, who thanked the team for the evolution of the program and approved their use for the forthcoming clinical trial in Bamako, per the NIH-funded study grant.

**Table 4:** Final Malian adaptation of the DPP-P2P

Finalized PPD-Mali sessions	Content
S1 Welcome to PPD-Mali	Overview of PPD-Mali program and proven effectiveness of DPP-P2P
S2A Diabetes Prevention and Control	What is diabetes and small steps to prevent or control diabetes
S2B 50 Healthy Choices in Bamako	Review of 50 healthy choices participants can make to get started
S3 Engage your family	How to engage the head and other women in the household
4 A Move More	Why and how to become more active
4B Track your exercises at home	Demonstrations and booklet showing recommended exercises
5 A How to eat healthy	Healthy diet elements & demonstrations with PPD-Mali recipe booklet
5B Portion size and tracking consumption	Demonstrations on how to measure portion size
S6 Strategies for reducing portions	How to reduce and keep track of daily portions
S7 The Energy Balance	Importance of balancing food consumed with energy expended
S8 5 tips for eating away from home	How to avoid fats and oils in street food and at family celebrations
S9 Drink water, not soda	Why drink more water & reduce sugars in juices, sodas, beer, or tea
S10 How to have a healthy heart	Importance of diet and exercise for a healthy heart
S11 Getting more support to stay motivated	How to maintain support from family and friends for your lifestyle changes
S12: Staying motivated looking forward	How to continue to make progress in sustaining healthy

## Discussion

The formative research process used in developing our adaptation of the DPP-P2P program faced many challenges: conversion of the DPP materials from English to French to Bambara, modification of tools for delivery by low-literacy CHWs, expansion of interactive elements to the group sessions, use of pictorial communication tools, adaptation of the materials from the US to Malian context. With input from stakeholders, CHWs, medical officers at the CSCOMs, participants, and the research team, we iteratively adapted the session materials in three stages to attain an adaptation that the team and stakeholders assessed to be appropriate for the Bamako context and ready to be tested more broadly in a clinical trial. By involving the CHWs who would be delivering the program throughout the adaptation process, we and they learned how the sessions had to be adapted to enable them to implement the program as intended by CDC’s DPP-P2P program for CHWs.

A key adaptation was to convert the social support session to one devoted to the importance and strategy for involving the head of the family, along with women sharing cooking. Family support is paramount, because in Malian extended families, members eat from common bowls, with the adult women of the household taking turns cooking for the whole family. As was pointed out in the CHW focus groups, the program needed to be structured to elicit the support from the household and other women in the household. Participants said that the program’s stress on involving everyone cooking meals made them see a realistic way that they could change their diet. Participant feedback on the suggestions for ways to engage the entire household in making diet and exercise changes was considered key to steps they had already taken or planned for adopting the program recommendations. Our findings regarding the importance of eliciting family support are consistent with other DPP adaptation studies [31, 39, 40, 45, 46].

Consistent with other studies showing the critical role of CHWs and other members of the care provider team in encouraging self-care [47–49], the feedback from the participants indicated that they felt the program helped them better understand their role in managing their diabetes. For those who had been diagnosed with diabetes and received an initial set of recommendations from their doctor (as described in Garanet et al. [14] and confirmed through our own interviews with the CSCOM medical officers), the tailored DPP sessions exposed the participants to a new way of looking at their role in controlling diabetes. They learned how to prepare healthy meals on a budget, received specific tips on how to maintain a healthy diet away from home, and had the opportunity to try out exercises at home. The high level of attendance at the end of the month-long presentations demonstrates their interest in learning about self-care and prevention for themselves and their family.

While this study was designed to involve all appropriate persons in the adaptation of the DPP for use in Bamako, it is not without its limitations. Because it was an exploratory study emphasizing the opportunities to elicit feedback from participants, the sample size was very small, too small to permit analysis of the effectiveness of the adaptation. Those recruited were primarily female, so we were unable to assess how gender might influence response to the program. Also, because the participants were selected by the CSCOM medical officers as having diabetes, hypertension or being obese, any assessment of the potential effectiveness of the intervention is on prevention of diabetes among those at risk but control for those already diagnosed. However, it is the intention of our adaptation to promote both prevention and control of diabetes, as the behavioral changes emphasized by the program are appropriate for both, the impact is on prevention of diabetes case. Additionally, the pilot study was carried out over a period of four weeks, which is fast enough for feedback, but not fast enough for promoting some of the most important recommended behavioral changes, specifically for diet and portion size. Thus, we did not expect major changes in dietary patterns, and we did not weigh the participants or collect any biomarkers. Despite this limitation, we observed that after one month, participants reported making some behavior changes, particularly in their daily exercise habits. As in other studies, this change in exercise as a way of controlling diabetes may reflect a better understanding of the concrete steps they can take to achieve control [25, 47]. Even though our findings are only suggestive of potential changes, they reflect those of Baghaei et al. who found that illiterate diabetes participants were 8 times more likely to adopt self-care behaviors after receiving behavior change education sessions compared to those who are literate [50].

## Conclusions

The iterative process of adapting the DPP-P2P sessions and materials for use in low-income neighborhoods of Bamako, Mali was informed throughout by the CHWs who would be implementing the program. The feedback from participants further directed the team to make the program even more interactive and participatory. The participants’ approval of the overall program was evidenced in their hope that this program could be made available to a much larger population. Their comments and experience suggest that the next step’s clinical trial of the finalized Malian adaptation of the DPP-P2P modules with the additional pictorial elements, supported by the booklets and videos on preparing healthy sauces and exercising at home would have promise for promoting behavioral changes critical to preventing and managing diabetes risk among low-income Bamako residents.

### Electronic supplementary material

Below is the link to the electronic supplementary material.

Supplementary Material 1

## Data Availability

The datasets generated and analyzed during the current study are not publicly available due the small sample size and risk of breach of confidentiality but are available from the corresponding author on reasonable request.

## Abbreviations

CDC: Centers for Disease Control and Prevention
CHWs: Community health workers
CSCOM: “Centre de Santé Communautaire” (community health centers)
CVD: Cardiovascular diseases
DPP: Diabetes prevention program
DPP-P2P: Diabetes prevention program’s power to prevent
HPI Institute: Health Partners International Institute
NCD: Non-communicable diseases
NDEP: National Diabetes Education Program
NIH: National Institutes of Health
PPD-Mali: “Programme de Prévention du Diabète au Mali” (Diabetes Prevention Program in Mali)
SDM: Santé Diabète Mali
SSBR: Small Steps, Big Rewards
US: United States
