# Supplementary material for: Formative research to adapt the ‘Diabetes Prevention Program- Power to Prevent’ for implementation in Bamako, Mali
Source: BMC Health Serv Res. 2024 Jan 11;24:61. doi: 10.1186/s12913-023-10515-6 (PMC10785539; doi:10.1186/s12913-023-10515-6)
Supplement: Supplementary file 1 — Supplementary Material 1 [file 12913_2023_10515_MOESM1_ESM.pdf]

Focus Group Transcripts: English translations and summaries from the Pre-Test

Focus group transcripts: Koulouba CHWs

Introduction:

Now you have seen a little about what we propose doing here in Mali. We would like to discuss with you your opinions about how we could implement this in Mali. So, we have some questions to discuss with you along these lines, do you agree to participate and to have the discussion recorded? (All agree….)After consents, interviewer continues, saying now we want to get started.

Question 1: As you know, "Diabetes and Blood Pressure" can be hereditary or can affect us through our diet. We'd love to know what you know about these two diseases, let's start with Diabetes. Could you tell us what you know about Diabetes?
Interviewer: Please share with us what you have learned about this disease in your community, workplace, there is no right or wrong answer. These are just exchanges. We're planning a training to prepare community health workers like yourselves to implement the program, so we need your suggestions to be able to know where to start the process.

I'd like to talk, diabetes is a neglected disease, people don't pay too much attention to it, if I can put it that way. Also, as you said so well, this disease can be transmitted genetically, but also through the consumption of certain foods containing a lot of sugar or foods that are too fatty. These can be factors favoring this disease.

There are many cases of diabetes here. We consume the foods that cause this disease, but there are people who have a hard time understanding when they are told that certain foods can give them "diabetes," especially given that we don't do much exercise.

Interviewer prompt: Who else wants to add something? Does everyone understand Bambara?

What I want to add is the fact that a lot of people don't understand that this disease can be caused by food. Many people think that it's only hereditary that they can have this disease. There are many hereditary cases, but there are three types of "diabetes,1,2,3, and not all are inherited.

Interviewer: What do you know about high blood pressure? Is there anyone who wants to talk about "hypertension"?

They say that hypertension is a disease for the elderly, but we notice today that there are young people who have hypertension, which explains that it is not a disease of the elderly alone. Is it salt or is it due to other foods we eat?

Interviewer prompt: Who's going to add anything?

It is said that if you practice sports or if you deprive yourself of salt in the evenings, you will not have hypertension. We do not understand this because very recently a ten-year-old child was diagnosed at our CSCOM with hypertension. We would like to know how these diseases arise,

and what is the difference between those figures that go up and that go down when the doctor checks our pressure.

Interviewer prompt: Is there anything else you would like to add? The one at the end? As already said, there is no right or wrong answer, so do not worry if it might or might not be what we want to hear. We want to hear everything you know. I am here only to listen to you. So, we beg you to share with us what you do know. This will give us a better orientation on what to teach and it helps the project to move forward.

Well, tension is hidden in us, because we don't notice these sorts of things about ourselves. We may be sick, but it's only when we get to the hospital that we realize that it's "tension." We don't watch ourselves, we don't pay attention to our illnesses.

Interviewer: Thank you all for sharing your views. This has been very helpful. Now I would like to move to the next question.

Question 2:

Do you know anyone with diabetes? Do you have this disease? Do you know anyone who has gone to the hospital because of this disease? Do you know people who have died because of Diabetes or Hypertension?

My aunt has diabetes, she does not eat sugar anymore. But there is another manifestation of a rash on her buttocks, and I wonder if this is due to diabetes or not?

I have an uncle developed tension, even though he was doing sports and everything. He was a teacher. He had a stroke, and to this day his left side is dead, and he has been advised not to consume ordinary salt, only a small amount of sea salt.

It's only when a patient comes back from the hospital, after the tests, that we know that a person has diabetes. So, we'd like to know the signs that predict diabetes?

Interviewer: As already said, we only collect what you know about these diseases today. It is through your testimonies and experience that we will develop the project.

Diabetes runs in my family, my uncles and aunts have it; I have parents who have had their feet cut off. My dad has diabetes, and he frequently has tests to do. I have blood pressure and every time I go to the clinic, I do tests for diabetes, because I know that it is in the family. The signs that predict it in us are the pimples that appear on the body, anyone who has experienced this has ended up having diabetes after the tests. If I see any pimples on my body, I stop consuming sugar.

There are many people who have suffered from this disease. My grandmother has high blood pressure and diabetes at the same time. I also have an aunt developed diabetes, but she was ashamed to say that she is diabetic. Unfortunately, the doctor who received her did not try to find out her sugar levels before prescribing a glucose serum, and she lost her life.

There was a person in the family who died because of tension, and I know people who had limbs cut off because of diabetes. Others lost their lives.

Interviewer: Thank you all for these testimonies.

Question 3:

Interviewer: Now, we are going to discuss the following question:

Research has shown that tension and diabetes can be prevented, and those who already have it can reduce how serious the illness is if they change their behavior. In your opinion, what behaviors can prevent or lessen diabetes or tension? What can we do about these diseases?

You can change your behavior with food when you know you have this disease; there are things you won't eat. You can do physical activities. This can lessen how bad the disease is.

People living with diabetes may be advised not to eat from 4 pm. onwards. If these people can do without dinner, it would help them more.

People living with diabetes might be advised to stop eating white rice and instead eat the smoked rice "malowusu" Also, you are forbidden to eat sweets.

We would advise you to reduce consumption of sugar, and decrease consumption of certain foods. Sugar is reduced for diabetes and salt for "blood pressure".

Young people who have tension can do sports and old people can set their sugar aside.

There are medications for diabetes and blood pressure that are taken on a daily basis.

A lot has been said about sport, but there is also "obesity" which is a factor that promotes diabetes. For diet, a suggestion is not eating from 7 p.m. until the next morning at 8 a.m.

Question 4:

Interviewer: Thank you! *Now, you've all talked about behaviour change, but you know as well as I do that change is easier in theory than in practice.*
What can promote behaviour change in people with diabetes or high blood pressure? For example, if a person with high blood pressure is advised not to eat salt, what can help them adopt this new behavior?

This change can be easy or difficult, depending on how you approach the person, you have to help them understand the seriousness of the disease, but at the same time reassure the person that there is a solution that can allow him to live with this disease, namely the recommended behavior change. For me, the right explanation can make it easier for the patient.

Each of us has to learn to avoid certain foods that cause us discomfort or make the disease worse. The doctor can't tell us all this. For example, if you feel discomfort after consuming sugar, it's up to you to be able to identify this and find a solution.

Interviewer: Thank you! Who else wants to add something? How can we help facilitate behaviour change in patients?

We have to learn how to tailor the change recommendations to each person. All these changes in behaviour that we have just put forward can reduce the effect of these diseases, but we can't suggest the same method of change for everyone. For example, we are talking about sport, can a 60-year-old person do sport? It's difficult. We also talk about changing food, you will find that there are people who can barely afford to feed themselves, so if we were to suggest to the latter to change his diet we risk creating more difficulties for them. It will be seen that each person has his or her own reality, and we should tailor the advice is given to each person.

I think it depends on each individual because others are more resistant to change than others when it comes to depriving themselves of certain foods or meals.

Interviewer: Thank you! Who else wants to add more?
It's not easy to deprive yourself, but whoever manages to start can continue.

Interviewer: So, how do you think we who are here can help them overcome their resistance to change?
After awareness-raising, it would be very good to involve a close person, this can be the spouse, a child, the latter can remind you repeatedly while encouraging you. It will be good if this person has the same information as the patient.
I think that in order for patients to listen to us, we need to have confidence in ourselves, When the patient sees that the doctor has confidence in the “relai,” it encourages the “relai” and in turn gives the patient confidence in the “relai.”

Question 5:
Interviewer: As she just said, it's up to you, the "relai". ***As a "relai", how do you think you can help them adopt this change in behaviour?***

If we educate the sick person, we must also educate the person who takes care of his or her food; otherwise, the patient can prepare what he or she wants and the patient who has no choice will eat what is offered.

Interviewer: If I understand, you have to build trust and educate the patient <u>and</u> the family member who prepares food for the family.

My intervention follows on what she just said. Often, the doctor is far away but the "relai" is in the patient's neighborhood or may know a close relative of the patient. Then, the "relai" can take the opportunity to explain and educate the patient's relatives by explaining the things that the doctor has not had time to explain.

As a “relai,” we have to be patient in giving explanations, whether the patient is an acquaintance or not, we have to support the patients.

Interviewer: [participant’s name] and your neighbor, would you like to add something as a "relai" that you can do to support people with "diabetes and high pressure" to change their habits?

As a "relai", after educating the cook, we can also tell the patient, who may spend the day in bed, to try move around very often or at least to have his foot massaged if he is unable to move. In addition to this, it is good for the patient to speak and have conversations with their loved ones. When you are called, you have to have the courage to speak softly.

Interviewer: Thank you! How do you think we could help you make it easier? As you said before, you can help them, how can we help you?

I think you need a high-quality training. [the project training director] said somewhere that the "relai" must be confident, because that in turn gives the patient confidence in the “relai”. The "relai" plays the role of the doctor in explaining the intake of the medication. Because many questions can be addressed to you, the "relai, you must be able to answer these questions.

What I meant is that a person must have confidence in himself, that's how the one we are addressing will trust us. When the sick person is well received, he relaxes and can be enlightened. Everything depends on the reception.

Interviewer: Your words complement each other. When you receive the patient with confidence, the sick person will be comfortable asking you questions, and you must know how to answer him.   The "dogonomusu" and her neighbor, do you have anything to add?

Often when we go to raise awareness, there are people who ask us if we have any medication to give them. If you explain their illness to them they don't listen to you.
Interviewer: If they ask you if you have any medication, let's say you are asked to go talk to a person with diabetes, what can you do as a "relai" so that they adapt the behavior change?

You're going to explain it to them, you also ask if they're already taking a medication because there are some people who don't know that they have blood pressure or diabetes because it can't be controlled, but those who are already taking a medication, we can better guide them on how to take the medication.

It depends on the explanation you are going to give them, making them see the benefit of the change in behavior that can save them from taking a lot of pills.

Question 6*:*
Interviewer: *You know that most people eat as a family, so how can we encourage changes on behalf of one of the family members, for example to cut down on bouillon cubes*?   *What do you think would happen if these people proposed to their family to decrease their use of bouillon cubes? It might help to point out that the entire family could become diabetic or hypertensiv.,*
So the question is: what can the "relai" do to help the family adapt their eating habits?

It's a bit like what I said earlier, for example when you tell a woman to decrease her use of bouillon cubes , she will tell you that what she has as a condiment price is not enough, no matter what you say over and over again she won't change.

It comes down to the same thing, the bouillon cube problem is at our level, when you advise a cook from the big family to reduce use of bouillon cubes, she will answer you by saying that she prepares according to what she receives as a condiment price so, it becomes complicated when the change in behavior mixes with the price of condiment. The "Maggie Cube" problem is at the level of us women who need to prepare tasty food, otherwise all men complain about it.

Interviewer: I gave the example of bouillon cube, but you also gave many other examples like the one who has diabetes to reduce sugar consumption or the one who has tension to avoid salt. My question is that if they have to adopt a change with their family, what are the difficulties they will face in making that change?

It can lead to murmurs, family members may say, why do we have to change our meal because of one sick person?
Even if we were to try to enforce reduced use of the bouillon cubes, and substitute more smoked fish, there are people who hide that they are from putting in the "Maggie cubes" when no one sees them. Although everyone agrees not to put any.

Interviewer: We mentioned the difficulties, what can be the solution to the problem of condiment, murmuring... What can make it easier for families in this process of change?

They have to believe what they are told to make it easier to raise awareness. The whole family can't change in one go, it goes slowly on the basis of the one who adheres to this change, the people will adapt little by little, For example, when you bring a dish without a bouillon cube, you will find people who will fine it tastes ok, while others refuse.

Interviewer: So for her, change is coming gradually. Is there anything else to add? Otherwise, we continue. Very often when we talk about "sports", people think of great physical activities such as football, running and others, but it has been shown that there are easy and simple "sports" such as stretching in the house, walking. .. What sport can they start with if we advise them to do the sport for 15 minutes?

I think they have to start with the steps.
They can do cycling, whoever can't do it can work. K46: There are bicycle pedals that you can pedal while sitting without moving.

Interviewer 1: If we tell them to start with 15 minutes when they come back we suggest they go to 30 minutes, will they accept?

If we let him see the impact that a 15-minute month of sport has had on him, he would accept.

Interviewer: What helps them to dedicate 15 minutes of their time to sport?
Loved ones can help by reminding them.

Interviewer: What do you think can make the 15-minute activity difficult? we've talked about the difficulties of changing eating behavior, it's the turn of sport now, What makes it difficult for those with diabetes to move?

I think there are 2 things that can make these things difficult: occupation or laziness. There are people who work on site so it will be difficult for him to take 15min to do the sport, on the other hand there are people who do nothing but we are lazy.
Some have a sore body, others who don't have anyone to help them get around.
There are people who need help from a brave person to be able to move from time to time.

Interviewer: Now that you've talked about the difficulties, how can you convince people who are sick to exercise? As a "relai" how could you help patients to have the courage for sport?

To be able to help them you need gentle words, nowadays if you don't take something back several times people forget as a "relai" we have to have the courage to explain it repeatedly.

Interviewer: When we start, we will set up a group of 15 to 20 people per session, the nurses and the "relai" will come and lead the sessions by asking them about this patient and explaining to them that every week there is a commitment. For example, a patient may say this week I would not eat salt or sugar and would do 30 minutes of exercise. They will be in groups to support and encourage each other. ***What do you think we can do in the group to encourage them to the point where they are excited for the sessions?***
That can come from a very good explanation.
Interviewer: In turn, what can we do to get them to come to the session en masse and put the advice into practice?...

You have to train us well so that we can provide them with detailed explanations.

Interviewer: We would like to give them the information so that they understand so we would like to provide pictures for example you often see pictures that talk about diarrhea, pregnancies... Do you think these images will make your job easier?
Overall Answer: Yes
Yes, they will trust us more with images than with just words.
Yes, the images as well as an identifier that proves that we have been sent, because there are those who do not believe us without seeing it in writing.
Interviewer: What can this identifier be?
They want to see something that proves that we were coming from reliable agents, we have this problem during vaccinations they say we don't have an ID.
It can be badges.
We'd still like to have a written authorization in addition to the pictures, because when you get to people's homes they want to see the letter.
Yes, often when we go out with the medicine to give, they ask us if we have an authorization, and unfortunately these cases happened to us when we only had our vaccine coolers, no badge, no t-shirt, nothing.

Interviewer: We are going to provide you with picture brochures that the participants can also have, do you think this will matter? For example, they will leave with an image of the week that will remind them that they will not eat salt or sugar, sports time... Do you think we can also put it on their phones?
Answer to set: yes
Interviewer : Beyond that, we don't have funding yet, when we have funding, we would like to make videos that explain all these activities, do you think these videos will be beneficial for you and for them?
The answer to the whole: It will help us all.

Interviewer: We are now at the end of our discussion questions, so if you have any other information, suggestions, recommendations, to add, we will listen to you. Or if you have any concerns, questions about this project. Just one last word to close.

: As a final word, we kindly encourage you to train us.
: Well, the little I would like to add is that the initiative is good but even if we do find people with these diseases, they are often afraid to go to the hospital, there is a reluctance because they have financial difficulties so I propose support in this direction that will allow us to quickly convince them by saying that there will be a discount on the price of medicines.
If they can get support in terms of medications, we can motivate them by telling them that they will get a discount on the cost of medications because there are some who know they are sick, but they stay at home and use traditional medications because they don't have the financial means.
Interviewer : I get it. Anyone want to add something? ***If we offer you to take part in this project so that we can help them adopt a new behavior, would you be willing to participate?***
Collective answer: yes, yes
Interviewer: Thank you very much to all of you.

Focus Group Transcripts-Taliko CHWs

Interviewer: Thank you for agreeing to participate in this group discussion, and to have it recorded. Please be reassured that the chat we are having will remain between us, after writing we will delete all the recordings. I would like to begin now with the discussion.

Many people suffer from diabetes or hypertension in Bamako. These are not communicable diseases, but we all know that if this disease is in your family, you might have it. Beyond that, even if you don't have a relative who has diabetes or hypertension, there are behaviors that could make your more likely to get it. It is not an illness that can be cured in the blink of an eye. You will live with it, but there are things you can do to reduce its severity, such as for those with diabetes to restrict their rice consumption, to reduce their blood sugar levels. Those with hypertension are told not to do this or that. This advice is there for those being helped. So, as we said, the idea is to help people who have diabetes and hypertension in your community to change their behavior to strengthen their health.

As I told you, you play a crucial role; nothing can work without you. We would first like to know if people will like this program? Will they benefit? What are the possible problems that could arise? If we have your suggestions, we can improve the plan, so that it is based on what people like. We would also like to have your opinion on whether people will like it or not? Beyond that, how do you think we could spread the information and get people involved?
So let me get started with specific questions.

Question 1: What have you learned about diabetes in your community?
We thank you very much for bringing us together to educate us more about diabetes. What we have learned from the presentation on hypertension and diabetes is that if you get this disease, there is no cure; it brings a lot of small illnesses. There are medications that you have to take to survive. After understanding, we too could guide the people of our community. This will reduce this disease otherwise, hypertension and diabetes would make us suffer enormously especially for us who are the poorest.

I would like to talk about diabetes. I have diabetes. It is an incurable disease but if you follow the suggested diet, it can reduce the effect. Otherwise it is difficult. They give you measurements and medications to take monthly. I take insulin in the morning and evening. Well since my illness was declared I haven't eaten very much sugar.

Interviewer: Who wants to add?
I pray that we rise again in peace! I pray that Allah helps us in everything we do! The reason why we are gathered, may Allah help us in this sense. The information I learned about diabetes is that it is an incurable disease. I have a neighbor who had her foot cut off because of diabetes. Why? Because the foods she was told to avoid, she couldn't. In front of these children she pretends to have avoided them, but she asks for sugared tea to be made or other foods in their absence. As a result, a sore appeared on her foot, and finally her foot was cut off. All the people I saw with hypertension or diabetes never recovered from these illnesses.

Interviewer: Who wants to say something about "hyper tension"? What do you know about this disease? What have you heard about this disease in your community?

Before hypertension was for the elderly, but now I see in our community that children also have this disease, I cannot understand that there are more children than elderly people who have this disease.

Interviewer: She says that there are more children than old people who have this disease although this disease is known among the old. Who wants to say something more? As I already said, there is no right or wrong answer. It's as if we didn't know anything, we are here to learn from you, so we can then use this information for our program. For example, if in our program there were only old, what she has just said draws our attention to add the case of young people. Your answers give us ideas.

I know that there are two models of hypertension, hypo and hyper. If you have an increase in blood pressure, they advise you to avoid salt at night. If you have low pressure, they advise you to eat one quality of salt and avoid another. I didn't understand anything about tension because when you have it, there is a part of your body that becomes paralyzed, there is no solution in that you will spend the rest of your life with it. We seek to understand together with the help of God.

It used to be said that tension is a hereditary disease, but nowadays people are affected by this disease without any of their parents having been affected in the past. Others say that it is the massive consumption of salt that causes it. We cannot understand this disease. If the tension leads to a stroke while there is no one nearby, you risk losing your life or a part of your body will no longer be able to do anything. I have an aunt who is affected and so far her side is not working, she cannot do anything without the help of another person. When you go to the hospital, the doctors will tell you to avoid salt on from noon but you will see that there are people even after having followed this, their blood pressure rises after the analyzes. We often say that stress (dusukasi) increases tension for some people. We have just recently learned that the severity of the ulcer (fouroudimi) increases blood pressure in this case you need a prescription. Anyone who follows the instructions for taking medication and manages to stay away from the forbidden things will make their life with it. This is what I understood about the hypertension disease.

I did not understand how the tension manifests itself, why? I brought a pregnant woman in labor, she did all her assessment without hypertension but at the time of delivery we detected the hypertension This surprised me because they say that hypertension affects a person when you have a descendant who had this disease but that was not the case.

Question 2:

Interviewer: After your various testimonies I would like to know if anyone has gone to the hospital or lost their life because of these illnesses. What is your experience with these diseases?

I have a “co-wife whose dad had high blood pressure. At each visit he was told sometimes that the tension had increased and sometimes that the tension had decreased. We stayed like that until one day, he fell down [with a stroke] and that was the end of him. His daughter said he had tension but not the serious one. If you can find a solution to these cases, that is to say a place for the test, there are often people who die from it without realizing or even seeing a sign in themselves.

I learned that hypertension affects people who are fat but nowadays, thin people have this disease. I would like to understand this.

The interviewer: As I said, we are going to give you training to help you understand and be able to help the sick. Today we need your points of view.

I saw someone who had diabetes without knowing it. One day when we passed by her, she showed us a wound that was on her foot; it was a very deep wound. I advised her to go to the hospital, and not to ignore such a wound. Once she left, we detected that she had diabetes and found that the wound had affected her foot a lot. She had started these treatments at CSCOM before being transferred to Banatoukoro for the continuation of her treatment.

She was an acquaintance who had diabetes. She was under treatment, as she was leaving for work she had an accident and her ankle was injured. As she was diabetic the foot could not heal itself, to the point that we had to cut her foot. No more than three days later she died. Diabetes got the better of her.

With everything they have just said, I think your work is to be congratulated, it will be a very good thing for the community, for the country. Someone to talk about doing the test, I went with a friend to a campaign where we were offered to go for the test. The test declared that my friend had hypertension although all the doctors gathered around her, she did not admit it and as a result she did not buy the medicines that had been prescribed to her. After one or two years she fell ill with this illness. We told her to avoid salt, but she didn't want to. We must work to educate people to avoid the pushers of these diseases to survive. You are doing a good job, because there are many people who have this disease but refuse to admit it. It can affect fat people, for example people who have fat upper parts and thin lower parts. They could get a this disease in the future. When we tell them this they will not believe. But, it will be better to prevent it before it starts. Your work will be very beneficial for the country. May Allah guide you!

I didn't know that I had diabetes, because no one in my family had this disease. Before when we talked about diabetes people said that it was a disease of the rich, but we see that the poor are the most affected. I started losing weight and lost weight to the point that people asked me what was wrong. And, after that I was sweating a lot. I went to the maternity twice, and they said that when you are in menopause sweating helps you more. Then, I fell and was transferred to the emergency room for three days in a coma, then transferred to Banatoukoro for a week in the diabetes patient section. I would like there to be an assessment of hypertension and diabetes upon arrival of each patient, because many people live with this disease without

knowing it.   I did everything to find out, but I was told that it was menopause. Imagine if when I fell the doctor gave me a serum without doing blood tests? It could have been fatal to me.
Interviewer: Your interventions will be very useful to us because they enlighten us on many things. You know a lot about hypertension and diabetes.

Question 3:
Interviewer: Research has shown that we can prevent or lessen the effects of these diseases. These preventive methods include changing our diet, for example, by reducing our juice or soft drink consumption to reduce the risks, to consume less sugar. What are the behavioral changes that a person can adapt to either prevent or reduce the effect of these diseases?
The change can be to do without salt, sugar, take medicine when necessary if we receive this recommendation.
We must try to diversify our meals by reducing the consumption of salt. Cooks tend to put lots of salt-concentrated bouillon cubes in the sauce.

Interviewer: If I understand you, the change you are proposing concerns food, what other change can we propose?
What I suggest is to walk more until you sweat.

Before when you are worried about high blood pressure, we were told to reduce salt we consume during the day and to avoid all salt at night by only drinking porridge. Anyone who has high blood pressure and who continues to use salt regularly will eventually fall from a stroke. For diabetes, this disease has signs such as excessive sweating, peeing a lot. When you observe this it is the beginning, and then you have to pay attention to your sugar consumption.

We can change our diet especially at night, it is not good to eat or drink and then to go to bed immediately afterwards.

The change must involve your children and grandchildren.

We must avoid going to bed just after a meal; we must do some movement.

The interviewer: Change is easier in theory than in practice for a person. We think that change comes little by little, for example we won't say, don't drink drinks but rather reduce the quantity you consume until the person can completely stop. In your opinion, what are some small behavioral changes that we can suggest to someone with diabetes or high blood pressure?

We must reduce the oil and take long walks until we sweat.

The change that I propose is to reduce the consumption of two things in one to then half and finally you will do without it, this change must involve the one who prepares.

I think that counseling and monitoring can help.

Interviewer: Like what advice?
We explain to them the consequences, the limbs that can be cut...we don't force it but rather we give them advice about consequences that are scary.

People with diabetes have to take long walks. I am advised to walk up to 50 meters each morning, although I do not walk up to this distance. I do a lot of movement, the more you walk the more you can cut down your blood sugar. If you see that people with diabetes have a lot of discomfort on their feet, it's because of the lack of movement and the sugar that goes down into the foot becomes potash.

I'm not saying it's necessary but I would like to know how old men and women can get moving?

Interviewer: Do you have a proposal to make to us? We are here to exchange ideas.
I think we need to go to the family heads.

Yes, I think we need to go see the family heads and explain to them, because even if we say to reduce sugar this may not apply to everyone. Here in Africa it is frowned upon to cook your own food apart from others. But if the family heads are aware it will allow you to have your own cooking, or else that the quantity of salt or sugar will be reduced for everyone. I think this could be a solution.

Question 4:
Interviewer: What can we do to help them adapt to certain changes? As we said, change comes little by little. She just mentioned that we can go see the heads of the family, take walks, reduce salt or sugar. We are going to bring together people who can benefit from making these changes, but how can we make suggestions for change? What can make this easy for them? **What difficulties do you think people with diabetes may have in adapting to change with their family? For example, when you advise someone to cut down on bouillon cubes, what can be hard for them?**

Collective response: There are many difficulties
Interviewer: let's talk one by one.

It is very difficult if I am advised not to eat salt, since I eat with others. I risk making it hard for them.

It's very difficult. Suppose you are advised to stop using bouillon cubes. But, you live with someone who cannot prepare without using these cubes. The cook will think that the person who wants change wants to hurt her reputation. What I propose as a solution is to join forces with the one who can impose themselves in the family.

I think one of the difficulties may be the money problem. If you eat with everyone and now you have to eat alone, you will have to cover the costs yourself. We can also advise the head of the family to think about buying things for the sick person who cannot eat salt.

Interviewer: What can make the task easier for the patient?
We are going to talk with the cousins because the health of a family is in the hand of the cousins. The task will be easier for us if they agree to cooperate.

The interviewer: Let us talk about sports. You have already highlighted walking by saying that it can reduce diabetes. Very often when we talk about sport, people tend to think of soccer or running. Now, we would like to suggest a sport which is very simple, such as stretching, walking. Or, putting on music and dancing, which we think is only for young people. Or play with your grandchildren just to have a bit of relaxation. So, if we ask the person with diabetes to do these kinds of sports in 15 minutes, what sport can they start with?

Walking for a few minutes

They can walk in their living room from one end to the other, counting the minutes. They will see that after only a little time, they will sweat.

The most difficult is for those who work in offices, spending the whole day sitting, especially if they go to bed just after eating when they get home.

You can lie down and swing your feet like this, this movement is good. I turn on my TV at 5 a.m.; at that hour there are exercises on Ivorian TV, so I try to practice everything they do while in my living room. At the end I sweat a lot. Just after that, ORTM also has its sports program.

Interviewer: As you said, it is not easy for everyone to do exercises. Do you think that when we tell them to exercise for up to 30 minutes, will they accept?

If they are well sensitized to the point that they start, we can start gently little by little until their body is used to it. Once the habit is established, their body will demand it.

I have a friend who didn't know she had hypertension until after giving birth. She felt better when she followed my advice to walk around the big school every morning.

Interviewer: So, what more can make it easy for them?
If you have the courage you can go with them. I have a friend who suffered from diabetes, the nerves in her foot became dry. Each time I travel, I invite him to go there on foot. We leave and return from Lafiabougou on foot. To this day she herself has a taste for walking on her own.

Even though my daughter-in-law is there and could do it, I choose to sweep the yard myself, the front of our house and then to put the water on fire. When I finish all that, I go to the market to buy the condiments for the sauce. I do all this so I can live in peace.

Interviewer: What difficulties might they have to exercise 30 minutes?

The problem is time, in this case, we can offer them to do it during the weekend.

There are some who are afraid of getting hurt when doing sport. I have a friend who refuses to accompany me for fear of scratching herself or hitting a stone. You will say everything but she will never go.

Question 5:
What can you as "relai" [community health worker] do to help them?
If it is a parent who has the disease, you may find that there is a child the parent listens to more. So, if we approach this child he will be able to make his parent aware. In any case, we must go into the families to give them information, during this visit, we will be able to determine if there is a child that a parent will listen to.

Interviewer: As we said before, how can we help you help them? You said to go talk to the head of the family. As a "relai" what can you do to help them adopt change?
As a "relai," we can go to these families to talk with them. There are people who do not listen to their family members, but if they know that it is doctors or their representatives who have come to speak they can listen.

I wanted to say what my mother said, that the work of the "relai" is to raise awareness. I can even say that we have already started to reach out to people. Only awareness can remedy this scourge.

She says the truth, We cannot do "relai" work well without raising awareness. We can't do this without your help, we need you to train us so that we know what to say, and also that we know that we didn't stand up on our own. We need to have things like badges, T-shirts and more that prove we work with people.

The interviewer: You community health workers of Taliko, it's as if you read my thoughts, you have just answered the question which should follow immediately.
Question 5:
How we can we help you to help them? She said a lot, who else has anything to add?

Collective response: She said it all

As a "relai" we played a big role in the eradication of malnutrition in Taliko. We follow each child who is malnourished until the child is doing well. We have done more than 5 months on this initiative. When you are a "relai" you need a lot of courage. We are very well known in and around our community. Even the children know us because we are in every family.

It all depends on courage, we cannot guide a person through change and the forgotten, we will have to be frequent with the person to help them remember.

The facilitator must speak in Bambara so that everyone understands, he/she must also know how to enliven it so that the talk does not become boring. When we do our talks instead of tontines on Mondays and Tuesdays, we are not long, we tell them the essentials, and then it is up to them to lead the debate. The presenter must be well dressed, with the body entirely covered, and respectful to the neighborhood. We do our talks in this sense to the point that he asks us in the street on our next visit. Our wish is the good health of the people who live in our community, so we will do our work to keep a brave heart.

Interviewer: The World Health Organization has developed pictures that people can adapt. I know you saw some of these during your trainings. Do you think these images will be useful during group sessions? Could you work with them? Would the members of your groups like these images? We can also make a book that they can take home. Do you think it will help them when we put these images on their phones?

Collective answer: Yes it will help them a lot, the images too.

The interviewer: the last question before you are given the floor. We don't have any money yet but we would like to schedule the editing of a video that can explain all the activities. Do you think these videos will work?
Collective answers: yes
Interviewer: Would they be willing to put them on their phone?
Collective rest: yes, if it pleases God, they will accept.

The interviewer: We will work with all you have said. We will try to insert your advice on reducing use of the bouillon cubes and the other suggestions. We will group people into groups of 15 to 20 people so that it is easy for them to exchange and learn from each other. You and the nurses will be the facilitators of these chat groups after we have trained you. As you know, when people get together friendships are formed, so they can remind and encourage each other in the neighborhood. The question for you is: What can we do to get them excited to join the group?
Make the group fun. Anyone who likes it will always come back, they will be able to say I'm going to hurry so as not to miss the story from the other day. They will be able to motivate themselves like that.

I would like us to bring people from Taliko together at the Taliko hospital. This may be different from visits to families. When we go to Banatoukoro we try to share experiences.

We are all recognized in our neighborhoods, people recommend us during vaccinations.

It's true that the work of a “relai” is voluntary, we are not paid, we want to help our community, but whatever happens we have to think about motivation, when we go into the field we have to think about the price of the water for us to refresh. We do not minimize an amount.

Question 6:
Interviewer: *As time is running out we will move on to the next question. This question concerns you all.* ***We would love to help you help people in your community. Are you available to join this initiative?***
Collective response: Yes, we all have time, which explains our presence here.

The interviewer: We are at the end of our interview so if you have anything to add, the floor is yours.
We liked it. If you do things as you said, it will help a lot for those with diabetes and high blood pressure and also rest assured that you will have enormous satisfaction with our team as we follow the guidelines.

The interviewer: Thank you everyone.

FGD Potential Participants- English Summaries of feedback sessions in Koulouba and Taliko

Feedback from the participants in the Pre-Test

- Welcomed the initiative for addressing diabetes and hypertension, because there is nothing like it and it will greatly help the community. The welcomed it so much that everyone wanted to talk about how great this would be. Had to stop the congratulations in order to move on.
    - According to them, the key is the importance of involving those with the illness and those without the illness in the same family. This way you will find a preferred opening into the family. But also it is important to engage anyone who is responsible for preparing meals.
    - Changing the family diet is difficult and always a sensitive issue, particularly with the example of the Maggi cube, which is difficult not to use as it is a matter of taste.
    - Financial means will be a brake on attempts to change the diet.
    - Had to make clear that this is about taking medicines, this is about changing their behavior. At first they thought this was only to learn about diabetes, but then explained that this is about changing behavior. By the second session, they understood that this was behavior change, not medications.
    - In fact, the program had too many who wanted to participate and we had to limit the number to 20.

- From session 4 the group focusing on physical activity recognized the low level of physical activity and felt it could be explained by lack of information and the already busy daily routines. Many had not thought how they could integrate exercise into their daily activities.
    - One participant said her only leisure was to watch television for specific programs.
    - But others commented that they had started to walk at least 15 minutes daily.
    - At Taliko they started a weekly walking group, identifying walking routes near schools, in the fields, along the avenues, and at Koulouba, they started exercise/work-out sessions on the roof of a house after seeing a video of the group in Taliko (see video)
    - Most stated that they would continue to increase their exercise levels themselves, pointing out that others would join them as they saw them being active.
    - They felt by the end of the sessions that they were moving more.

- Repeatedly the participants expressed interest in a program aiming to make sustained changes to their life style. They liked the messages and suggested strategies that were introduced by the facilitators.
    - They retained the key message to eat less fat and to walk, but added that it was important to have advice and that they would be supported by the CHW in facilitating lifestyle changes.
    - They were able to reduce salt in their food during the month.
    - They agreed on the principle to strongly involve the household head and the family, to discuss with everyone the changes they want to make to their diet.

- The eat-out module needs to be modified to encourage eating at home instead of everyone going their separate ways. They suggested we propose pooling the money so they can prepare a healthy meal.

- The participants agreed that at the start they thought such changes would be impossible, but with the sessions they saw and they tried it themselves. They could see they wanted to make these changes and realized that they could change the way they prepare food.
- The participants equally explained that they understood much more thanks to the explanations and teaching style of the facilitators. They now understood well that changes would be gradual, step by step. The obstacles would be their own laziness as well as their work. But being in a group would make it easier, as changes are hard when alone.

- Feedback on the style of the program:
  - Role play was easier to involve people, than the group discussions which took longer to organize.
  - Also presentations should avoid getting in the habit of reinforcing stereotypes of traditional roles.
  - Trying things out in the group was a big help, all agreed.
  - In order to support them in making changes, the CHWs need to be well trained and supported. Participants liked the pictures.
  - It would be good to have communications from opinion leaders supporting the changes, e.g. from religious and traditional community leaders whose influence can facilitate the adoption of these healthy behaviors in the community.
- **Summary of the major themes emerging from the feedback:**

  - Importance of the approach to the family: At the suggestion of the program facilitators, participants had reached out to the family head, and they had to learn how to address criticisms or questions from him and from others in the family. Managing these communications was considered a key to success. Everyone agreed that the women said they were not the only cooks, and they had to involve their co-wives. Participants had to learn to continue, ignoring negative criticism so that they would not become discouraged. It is always important to remind oneself and others in the family that the objective is your own health. This was good to note, as some members of their families were reluctant to make changes, particularly in their diets.
  - A big concern shared by many is how to modify the diet without increasing the cost of feeding the family. It was proposed that one could cut the cost of purchases, such as the Maggi Cube, which would allow this money to be used for vegetables to put in the sauces. However, it was repeatedly noted that any change requires the approval of the family head, who will certainly not increase his contributions. They agreed that the CHWs could give them advice on how to prepare healthy food without additional costs.

Another suggestion was to change snacking so that instead of everyone purchasing lunch or snacks separately, they could pool the money and use it to buy vegetables instead of Maggi cubes.

- Cost is not the biggest obstacle: One participant noted, " It is not so much a matter of cost as habit." They questioned how to apply the recommendations for portion size and calories when often meals are served from a shared bowl. There was a consensus that changing the content of collective meals requires group solidarity, so that habits are changed for all and for a long duration.
- Controlling diet is a continuous struggle but not impossible. We observed that the participants insisted on the importance of the Maggi cube. But they also now understood the importance of dietary diversity, with a better balance. They were also pleased to learn about good alternatives to rice, such as the traditional millet paste or le tô malien which has been abandoned by many with modernization.
- Changing physical activity: All agreed that this was much easier to do and indeed they had started immediately with this. Some had also started walking, by themselves or with their husbands. Some had already started to feel the good results of regular exercise. The pre-test groups had started walking and dancing groups for every Thursday (See vidéos Taliko et Koulouba). They suggested that we should find a coach to mentor these groups in their exercise plans so that their activities are more effective.
- The Game Plan: They liked the idea of a game plan (Un plan d'action), because it will help them continue to work for good results.
- Importance of the CHWs: More globally, the participants congratulated the CHWs who they say can and will come to them to explain and encourage them in making changes. Some had already started talking with their neighbors and friends about making changes. Their hope would be for the project to reach a large population, not just a select few.

Conclusion

The participants congratulated us for taking the initiative to develop the project, as they think this program will be of great benefit to their community. Until now, no other project has focused on non-communicable diseases and they know that something must be done.
